# Supplementary material for: Content-rich biological network constructed by mining PubMed abstracts
Source: BMC Bioinformatics. 2004 Oct 8;5:147. doi: 10.1186/1471-2105-5-147 (PMC528731; doi:10.1186/1471-2105-5-147)
Supplement: Additional File 2 — The original results of the above study (non-essential files are deleted to keep the file size under the limit set by BMC bioinformatics). [file 1471-2105-5-147-S2.bz2 › chilibotAdditionalFile2/dip30/386ID10318834E9856/html/EPO_EPOR.html]

 


 **EPO** and **EPOR** 
  
Found 716 abstracts in PubMed, retrieved 30.  
 

 What does Google say? 
 PDF only 
| .edu only 

---

**Interactive relationship** (e.g. stimulation, inhibition, etc)

**Neutral relationship**- Both  **Epo**  and  **EpoR**  are expressed by astrocytes and neurons and  **Epo**  is present in the cerebrospinal fluid CSF.  Ref: 12638727 J Neuropathol Exp Neurol, 2003
- Dimerization and tyrosine phosphorylation of  **EPOR**  were found after 1 minute incubated with  **EPO** .  Ref: 12578614 Zhongguo Shi Yan Xue Ye Xue Za Zhi< MedlineTA>Zhongguo Shi Yan Xue Ye Xue Za Zhi, 2003
- Cytoprotection by  **EPO**  is initiated by the activation of the  **EPO**  receptor  **EPOR**  and subsequent signal transduction pathways that originate with the Janus tyrosine kinase 2 Jak2 protein.  Ref: 12769640 Curr Drug Targets Cardiovasc Haematol Disord, 2003
- Erythropoietin  [ **EPO** ]   **EPO**  regulates mammalian erythropoiesis by binding to its transmembrane receptor  **EPOR** .  Ref: 12878211 Biochem Biophys Res Commun, 2003
- Several reports have suggested an interaction between the erythropoietin  [ **EPO** ]  receptor  **EpoR**  and the shared signaling subunit hbeta c of the human granulocyte macrophage colony stimulating factor GM CSF, interleukin IL 3, and IL 5 receptors, although the functional consequences of this interaction are unclear.  Ref: 12488507 J Leukoc Biol, 2002
- A characteristic cluster upregulated in the  **EPO**  R  [ **EPOR** ]  and  **EPO**  W O groups and downregulated in the  **EPO**  H group was identified that contained various cytokines and growth factors, including IL 6, GM CSF, vascular endothelial growth factor B, IL 9, IL 3, leukemia inhibitory factor, and interferon alpha 2, and such receptors as thrombopoietin receptor, IL 9 receptor, and colony stimulating factor 1 receptor.  Ref: 12612986 Am J Kidney Dis, 2003
- These findings indicate the expression of multiple  **EPOR**  isoforms in human cancer cells that may modulate the cellular effects of recombinant human  **EPO**  or  **EPO**   **EPOR**  antagonists.  Ref: 12878211 Biochem Biophys Res Commun, 2003
- The multifunctional role of  **Epo**  has further been confirmed by the discovery in the central nervous system of a specific  **Epo**   **Epo**  receptor  **EpoR**  system.  Ref: 12638727 J Neuropathol Exp Neurol, 2003
- We conclude that in Chinese hamster ovary cells,  **Epo**  activates MAPK via a novel pathway dependent on G i association to  **EpoR** , G beta gamma subunit, Ras, and PI3K.  Ref: 12538595 J Biol Chem, 2003
- Signals provided by the  **Epo**  receptor  **EpoR**  are required for erythroid development beyond the CFUe stage, and are propagated via the  **EpoR**  tethered Janus kinase, JAK2.  Ref: 12869513 BloodBlood, 2003
- Presently, the activities of a PY null  **EpoR**  HM form in primary progenitor cells from knock in mice were further assessed using optimized  **Epo**  dose dependent proliferation, survival and differentiation assays.  Ref: 12869513 BloodBlood, 2003
- Using different in vitro systems, we demonstrate that 3 alternative pathways independently lead to  **Epo**  induced activation of PI 3 kinase and phosphorylation of its downstream effectors, Akt, FKHRL1, and P70S6 kinase through direct association of PI 3 kinase with the last tyrosine residue of the  **Epo**  receptor, through recruitment and phosphorylation of Gab proteins via either Tyr343 or Tyr401 of the  **EpoR** , or through phosphorylation of IRS2 adaptor protein.  Ref: 12506011 Blood< MedlineTA>Blood, 2003
- Strong evidence indicates that erythropoietin  [ **EPO** ]   **Epo**  is eliminated via  **Epo**  receptors  **EpoR** .  Ref: 12750427 J Pharmacol Exp Ther< MedlineTA>J Pharmacol Exp Ther, 2003
- The impaired cooperation between the Src defective Kit mutant and  **Epo**  R  [ **EPOR** ]  was associated with reduced transphosphorylation of  **Epo**  R  [ **EPOR** ]  and expression of c Myc.  Ref: 12486028 J Biol Chem< MedlineTA>J Biol Chem, 2003
- Deletion of this region in  **EpoR**  abolished both MAPK and PI3K activation in response to  **Epo** .  Ref: 12538595 J Biol Chem, 2003
- There was  **EPOR**  expression and the cell proliferation could be stimulated by  **EPO**  in KOCL 33 cells.  Ref: 12578614 Zhongguo Shi Yan Xue Ye Xue Za Zhi< MedlineTA>Zhongguo Shi Yan Xue Ye Xue Za Zhi, 2003
- Our results suggest that dimerization of  **EPOR**  and tyrosine phosphorylation were appeared under the stimulation of  **EPO** , and SHC and PLC gamma play a cooperative effect in  **EPOR**  mediated signal tranduction.  Ref: 12578614 Zhongguo Shi Yan Xue Ye Xue Za Zhi< MedlineTA>Zhongguo Shi Yan Xue Ye Xue Za Zhi, 2003
- Combined stimulation with  **Epo**  also resulted in impaired cooperation between Src defective Kit mutant and  **Epo**  R  [ **EPOR** ] , and to a lesser extent with Kit mutants defective in the activation of PI 3Kinase or Grb2.  Ref: 12486028 J Biol Chem< MedlineTA>J Biol Chem, 2003

**Non-interactive relationship** (e.g. studied together, co-existance, homology, etc.)

- RESULTS We detected  **Epo**  and  **Epo**  R  [ **EPOR** ]  localized within glandular epithelial cells in both peritoneal endometriosis and eutopic endometrium.  Ref: 12525458 Hum Reprod, 2003
- RESULTS The presence of  **EPO**  R  [ **EPOR** ]  was demonstrated on islets regardless of species.  Ref: 12717230 Transplantation, 2003
- Furthermore, administration of  **EPO**   **EPOR**  antagonists delayed the growth of uterine, ovarian, and mammary carcinoma cells in experimental animal models.  Ref: 12878211 Biochem Biophys Res Commun, 2003
- In order to investigate the expression of erythropoietin  [ **EPO** ]  receptor  **EPOR**  in leukemic cell line and clarify the mechanism of proliferative signal transduction mediated by  **EPOR**  in the leukemic cell line KOCL 33, biotinylated  **EPO**  and flow cytometry were used for detection of  **EPOR**  expression, 3 H TdR incoporation for cell proliferation, immunoprecipitation and Western blot analysis were used to investigate the tyrosine phosphorylation of signal transduction proteins as the causation of enhanced proliferation of KOCL 33 cells.  Ref: 12578614 Zhongguo Shi Yan Xue Ye Xue Za Zhi< MedlineTA>Zhongguo Shi Yan Xue Ye Xue Za Zhi, 2003
- CONCLUSIONS This is the first demonstration that pancreatic islets express  **EPO**  R  [ **EPOR** ] .  Ref: 12717230 Transplantation, 2003
- n = 10, rHuEPO responsive non HD patients with CRF  **EPO**  R  [ **EPOR** ] .  Ref: 12612986 Am J Kidney Dis, 2003
- **Epo**  receptors  **EpoR**  have been found on gastric mucosa and intestinal mucosa, and in mesenteric vessel endothelium.  Ref: 12477262 Acta Paediatr Suppl, 2002
- As compared to the wt  **EpoR** ,  **EpoR**  HM activity was compromised several fold in each context when  **Epo**  was limited to physiological concentrations.  Ref: 12869513 BloodBlood, 2002
- In this study we correlated microvascular density and  **Epo**  receptor  **Epo**  R  [ **EPOR** ]  expression in endothelial and tumour cells with histopathological type in gastric cancer.  Ref: 12605644 Histopathology, 2003
- Remarkably, restoration of only the Src pathway in a Kit receptor defective in the activation of all early signaling pathways demonstrated a 50% correction in proliferation in response to Kit stimulation, and completely restored the cooperation with  **Epo**  R  [ **EPOR** ] .  Ref: 12486028 J Biol Chem< MedlineTA>J Biol Chem, 2003
- Mice that lack the expression of Kit or  **Epo**  R  [ **EPOR** ]  die in utero of severe anemia.  Ref: 12486028 J Biol Chem< MedlineTA>J Biol Chem, 2003
- We also demonstrated that  **Epo**  phosphorylated Lyn is associated with tyrosine phosphorylated PLC gamma 2 and PI 3 kinase in WT  **Epo**  R  [ **EPOR** ]  FDC P1 stimulated cells.  Ref: 12504103 Biochem Biophys Res Commun, 2003
- Based on a rational design where the extracellular domains of dimeric erythropoietin  [ **EPO** ]  receptor  **EpoR**  or gp130 were substituted with heterodimeric V H V L regions of anti hen egg lysozyme HEL antibody and  **EpoR**  D2 domains, the genes encoding the chimeras as well as a model transgene, enhanced green fluorescent protein EGFP, were retrovirally infected into IL 3 dependent Ba F3 cells followed by direct HEL selection in the absence of IL 3.  Ref: 12655020 Nucleic Acids Res, 2003
- The role of these pathways in Kit induced growth, proliferation or cooperation with  **Epo**  R  [ **EPOR** ]  is not known.  Ref: 12486028 J Biol Chem< MedlineTA>J Biol Chem, 2003
- In Chinese hamster ovary cells expressing  **EpoR** , the G i inhibitor pertussis toxin blocked mitogen activated protein kinase MAPK Erk1 2 activation induced by  **Epo** .  Ref: 12538595 J Biol Chem, 2003
- Our results suggest that increased expression of  **Epo**  and  **EpoR**  may play a significant role in cervical carcinogenesis and tumor progression.  Ref: 12759237 Am J Pathol, 2003
- METHODS AND RESULTS Specimens of primary gastric adenocarcinomas obtained from 40 patients who had undergone curative gastrectomy were investigated immunohistochemically by using anti CD31 and anti  **Epo**  R  [ **EPOR** ]  antibodies.  Ref: 12605644 Histopathology, 2003
- We examined the role of the Src kinase Lyn in phospholipase C gamma 2 PLC gamma 2 and phosphatidylinositol PI 3 kinase activation in erythropoietin  [ **EPO** ]   **Epo**  stimulated FDC P1 cells transfected with a wild type WT  **Epo**  receptor  **Epo**  R  [ **EPOR** ] .  Ref: 12504103 Biochem Biophys Res Commun, 2003
- Acting via its receptor  **EpoR** ,  **Epo**  up regulates bcl 2 and inhibits apoptosis of erythroid cells and rescues neurons from hypoxic damage.  Ref: 12759237 Am J Pathol, 2003
- **Epo**  and  **Epo**  R  [ **EPOR** ]  expression levels within red peritoneal lesions were comparable to those of eutopic endometrium from patients with endometriosis.  Ref: 12525458 Hum Reprod, 2003
- **Epo**  s elimination kinetics was studied using a very sensitive tracer interaction method TIM before and after chemical ablation of BM as an indirect way of evaluating the  **EpoR**  through an assortment of PK parameters VM, KM, K, Cl used in differentiating the  **EpoR**  population in newborn and adult sheep.  Ref: 12750427 J Pharmacol Exp Ther< MedlineTA>J Pharmacol Exp Ther, 2003
- Stage IV gastric carcinoma had a higher degree of vascularization than other stages, and  **Epo**  R  [ **EPOR** ]  expression in both endothelial and tumour cells increased in parallel with malignancy grade and was highly correlated with the extent of angiogenesis.  Ref: 12605644 Histopathology, 2003
- METHODS Reverse transcriptase polymerase chain reaction, immunohistology, and Western blots were used to establish the presence and localization of  **EPO**  R  [ **EPOR** ]  on rat, nonhuman primate, and human islets.  Ref: 12717230 Transplantation, 2003
- It has previously been demonstrated that mutations in the 232 WSAWS 236 motif in the erythropoietin  [ **EPO** ]  receptor  **EPOR**  can result in strongly inhibited surface expression, due to defective intracellular transport Hilton, D.  Ref: 12859190 Biochemistry, 2003
- CONCLUSIONS  **Epo**  R  [ **EPOR** ]  level correlates with angiogenesis and progression of patients with gastric carcinoma and we suggest that  **Epo**  might have a trophic effect on the vasculature of the gastrointestinal tract.  Ref: 12605644 Histopathology, 2003
- Using immunohistochemistry, we examined the expression of  **Epo** ,  **EpoR** , p16, hypoxia inducible factor HIF 1alpha, and bcl 2 in benign and dysplastic cervical squamous epithelia and invasive squamous cell carcinomas ISCCs.  Ref: 12759237 Am J Pathol, 2003
- ...  **Epo**  R  [ **EPOR** ]  expression was significantly lower in black peritoneal lesions when compared to red lesions.  Ref: 12525458 Hum Reprod, 2003
- We showed that two inhibitors of Src kinases, PP1 and PP2, abolish both PLC gamma 2 tyrosine phosphorylation and PI 3 kinase activity in WT  **Epo**  R  [ **EPOR** ]  FDC P1 cells.  Ref: 12504103 Biochem Biophys Res Commun, 2003
- Assuming that  **Epo**  s elimination largely depends not only on erythropoietic  **EpoR** , but also non hematopoietic  **EpoR** , this work shows a significant difference in the relative proportions of the two  **EpoR**  populations in lamb and adult sheep.  Ref: 12750427 J Pharmacol Exp Ther< MedlineTA>J Pharmacol Exp Ther, 2003
- METHOD We investigated the localization of  **Epo**  and the  **Epo**  receptor  **Epo**  R  [ **EPOR** ]  in peritoneal endometriosis and eutopic endometrium, using immunohistochemistry.  Ref: 12525458 Hum Reprod, 2003
- In addition, adhesion of 32D  **EpoR**  Wt cells to immobilized H 296, a recombinant fibronectin peptide specific for alpha4beta1, induced tyrosine phosphorylation of Jak2, the erythropoietin  [ **EPO** ]  receptor  **EpoR** , and the IL 3 receptor beta subunit as well as Pyk2, Shc, and Cbl.  Ref: 12589802 Biochem Biophys Res Commun, 2003
- The G i protein constitutively associates in its heterotrimeric form with the intracellular domain of  **Epo**  receptor  **EpoR** .  Ref: 12538595 J Biol Chem, 2003
- These data demonstrate an essential role for Src pathway in regulating growth, proliferation and cooperation with  **Epo**  R  [ **EPOR** ]  downstream from Kit.  Ref: 12486028 J Biol Chem< MedlineTA>J Biol Chem, 2003
- The authors examined pancreatic islets for the presence of  **EPO**  R  [ **EPOR** ]  to determine whether these cells are protected by  **EPO**  from cytokine induced apoptosis.  Ref: 12717230 Transplantation, 2003
- We report an insight into the mechanism of commitment of erythropoiesis by activin A and BMP2 involving two key events, increase in  **EPO**  R  [ **EPOR** ]  and decrease in GATA2 expression.  Ref: 12531697 Exp Cell Res, 2003
- The proliferation and survival of hematopoietic cells is strictly regulated by cytokine growth factors that act through receptors of the Type I cytokine receptor family, including erythropoietin  [ **EPO** ]   **Epo**  and its receptor,  **EpoR** .  Ref: 12746455 J Biol Chem< MedlineTA>J Biol Chem, 2003
